# Evaluation of adherence to treatment in patients with anxious-depressive syndrome.

**DOI:** 10.1192/j.eurpsy.2024.1403

**Published:** 2024-08-27

**Authors:** T. Jiménez Aparicio, M. Fernández Lozano, M. Merizalde Torres, A. Rodríguez Campos

**Affiliations:** ^1^Psiquiatría, Hospital Clínico Universitario Valladolid, Valladolid, Spain

## Abstract

**Introduction:**

Treatment-resistant depression can pose a major challenge to mental health professionals, both in identifying cases and in devising consequent therapeutic strategies (1). However, it is not uncommon that the lack of response to antidepressant treatment is actually due to non-adherence to it in many cases (2).

**Objectives:**

In this context, it would be interesting to know the rate of abandonment of antidepressant treatment in patients with anxious-depressive symptomatology, since the patient’s evolution may depend entirely on this.

**Methods:**

To this end, the psychiatry service of the Hospital Clínico Universitario de Valladolid has collected data on adult patients who come for a first consultation in the mental health team, referred for presenting symptoms of anxiety and depression.

These data have been recorded over the last 2 years, including different socio-demographic and clinical variables. Subsequently, a descriptive analysis was carried out, the preliminary results of which are presented below.

**Results:**

We started from a sample of 222 patients at the present time: 69 men and 153 women, which is in accordance with previous data on the prevalence of anxiety disorders and depression by gender (3).

Antidepressant treatment was prescribed (from psychiatry or primary care) in 80% of them. A review 6 months later showed that up to 1/3 of these patients (34%) had abandoned treatment on their own before completing this period, as can be seen in the first graph (image 1), which is contemplated in several guidelines and recommendations in the scientific literature (4). No major differences were observed between genders for treatment indication or treatment abandonment.

On the other hand, 61% of the patients in the sample had been treated with benzodiazepines. Among them, up to 74% were still taking these drugs 6 months later (image 2). This result is striking, since in reality, the duration of treatment with benzodiazepines should be much shorter, according to the latest reviews (5).

Finally, cross-checking these data, it was observed that for 116 patients (52% of the total) the initial treatment included antidepressants and benzodiazepines. At 6 months, 18 of these patients (16%) had voluntarily discontinued the antidepressant, but continued with benzodiazepines.

**Image:**

**Figure:**
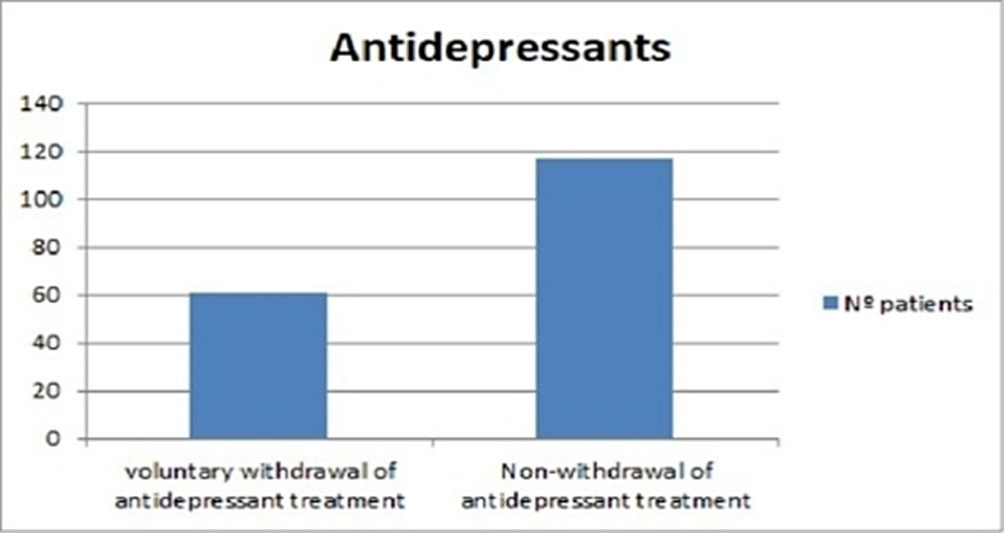

**Image 2:**

**Figure:**
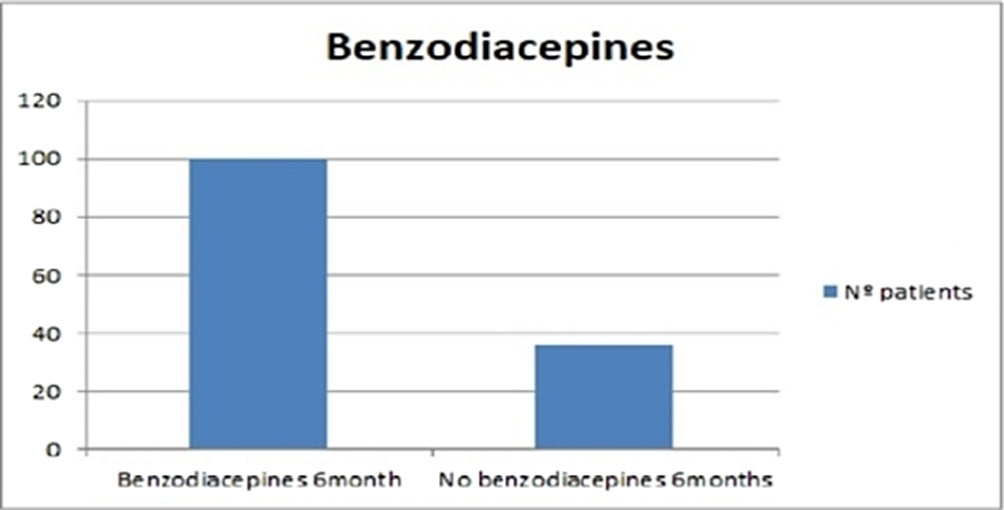

**Conclusions:**

It is very important to review adherence to treatment in all patients, especially in those cases in which the persistence of symptoms makes us think of a possible resistant depression. For this reason, it would be advisable to try to establish an adequate doctor-patient relationship that allows trust in the therapist and communication between both and leads to a favorable evolution.

**Disclosure of Interest:**

None Declared

